# Correction to: Complex systems of secrecy: the offshore networks of oligarchs

**DOI:** 10.1093/pnasnexus/pgad112

**Published:** 2023-04-19

This is a correction to: Ho-Chun Herbert Chang, Brooke Harrington, Feng Fu, Daniel N Rockmore, Complex systems of secrecy: the offshore networks of oligarchs, *PNAS Nexus*, Volume 2, Issue 3, March 2023, pgad051, https://doi.org/10.1093/pnasnexus/pgad051

In the originally published version of this manuscript, author Ho-Chun Herbert Chang's affiliation was incorrectly given as follows:

Annenberg School for Communication and Journalism, University of Southern California, Los Angeles, CA 90007, USA.

This has now been corrected to:

Department of Quantitative Social Science, Dartmouth College, Hanover, NH 03755, USA.

The Publisher apologizes for not correcting this error at an earlier production stage.

