# Supplementary material for: Complex systems of secrecy: the offshore networks of oligarchs
Source: PNAS Nexus. 2023 Feb 28;2(3):pgad051. doi: 10.1093/pnasnexus/pgad051 (PMC9998034; doi:10.1093/pnasnexus/pgad051)
Supplement: pgad051_Supplementary_Data [file pgad051_supplementary_data.pdf]

Supplementary Information for

Complex Systems of Secrecy: The Offshore Networks of Oligarchs

Ho-Chun Herbert Chang, Brooke Harrington, Feng Fu, and Daniel Rockmore

Ho-Chun Herbert Chang.
E-mail: herbert.hc.chang@gmail.com

This PDF file includes:

Figs. S1 to S2
Tables S1 to S3
SI References

1. Cumulative Table for Officer Types

The top 50 categories out of 651 total officer categories approximate 0.991 of all types. Beneficiary classes are highlighted.

Rank	Name	Count	Proportion	Cum. Sum
0	shareholder of	583438	0.344035	0.344035
1	director of	452535	0.266846	0.610881
2	secretary of	112192	0.066156	0.677037
3	judicial representative of	109526	0.064584	0.741621
4	legal representative of	99181	0.058484	0.800105
5	records & registers of	36318	0.021416	0.821521
6	auditor of	27242	0.016064	0.837585
7	**beneficiary of**	23602	0.013917	0.851502
8	managing director of	22624	0.013341	0.864843
9	**Ultimate Beneficial Owner**	21827	0.012871	0.877713
10	**owner of**	20400	0.012029	0.889743
11	director	20318	0.011981	0.901723
12	vice-president of	19676	0.011602	0.913326
13	liquidator of	17349	0.01023	0.923556
14	is signatory for	15626	0.009214	0.93277
15	president of	13495	0.007958	0.940728
16	intermediary of	8450	0.004983	0.94571
17	auditor	6371	0.003757	0.949467
18	**Beneficial Owner**	6047	0.003566	0.953033
19	secretary	6043	0.003563	0.956596
20	proxy of	5850	0.00345	0.960046
21	manager of	5127	0.003023	0.963069
22	treasurer of	4718	0.002782	0.965851
23	chairman of	3705	0.002185	0.968036
24	**beneficial owner of**	3010	0.001775	0.969811
25	appleby assigned attorney	2800	0.001651	0.971462
26	power of attorney of	2794	0.001648	0.973109
27	director / president	2694	0.001589	0.974698
28	**ultimate beneficial owner of**	2287	0.001349	0.976046
29	board-member of	1981	0.001168	0.977215
30	director / secretary	1905	0.001123	0.978338
31	executive director of	1442	0.00085	0.979188
32	trustee of trust of	1418	0.000836	0.980024
33	assistant secretary of	1362	0.000803	0.980828
34	president	1349	0.000795	0.981623
35	**partner of**	1348	0.000795	0.982418
36	connected to	1303	0.000768	0.983186
37	**trust settlor of**	1234	0.000728	0.983914
38	authorised person / signatory of	1229	0.000725	0.984639
39	chief executive officer of	1229	0.000725	0.985363
40	protector of	1228	0.000724	0.986087
41	chairman of the board of	1113	0.000656	0.986744
42	chief financial officer of	1110	0.000655	0.987398
43	power of attorney	1060	0.000625	0.988023
44	independent non executive director of	1055	0.000622	0.988645
45	supervisory director/commissioner of	1022	0.000603	0.989248
46	alternate director of	1019	0.000601	0.989849
47	president / secretary	1019	0.000601	0.99045
48	resident trustee of	999	0.000589	0.991039
49	local representative of	914	0.000539	0.991578

Table S1. Top 50 types of officer categories, which sum to 99.1% of all possible officers. Beneficiary classes are highlighted.

2. Sankey Diagrams

Sankey Diagrams by clients from each country, specifying their choice of intermediary, entity location, and offshore location. Shown are the top eight intermediary locations from each country. Russia's choice of intermediaries is much

more diversified than China, although China's choice of offhsore placement is much more diversified than Russia's. The United States in comparison is relatively balanced.

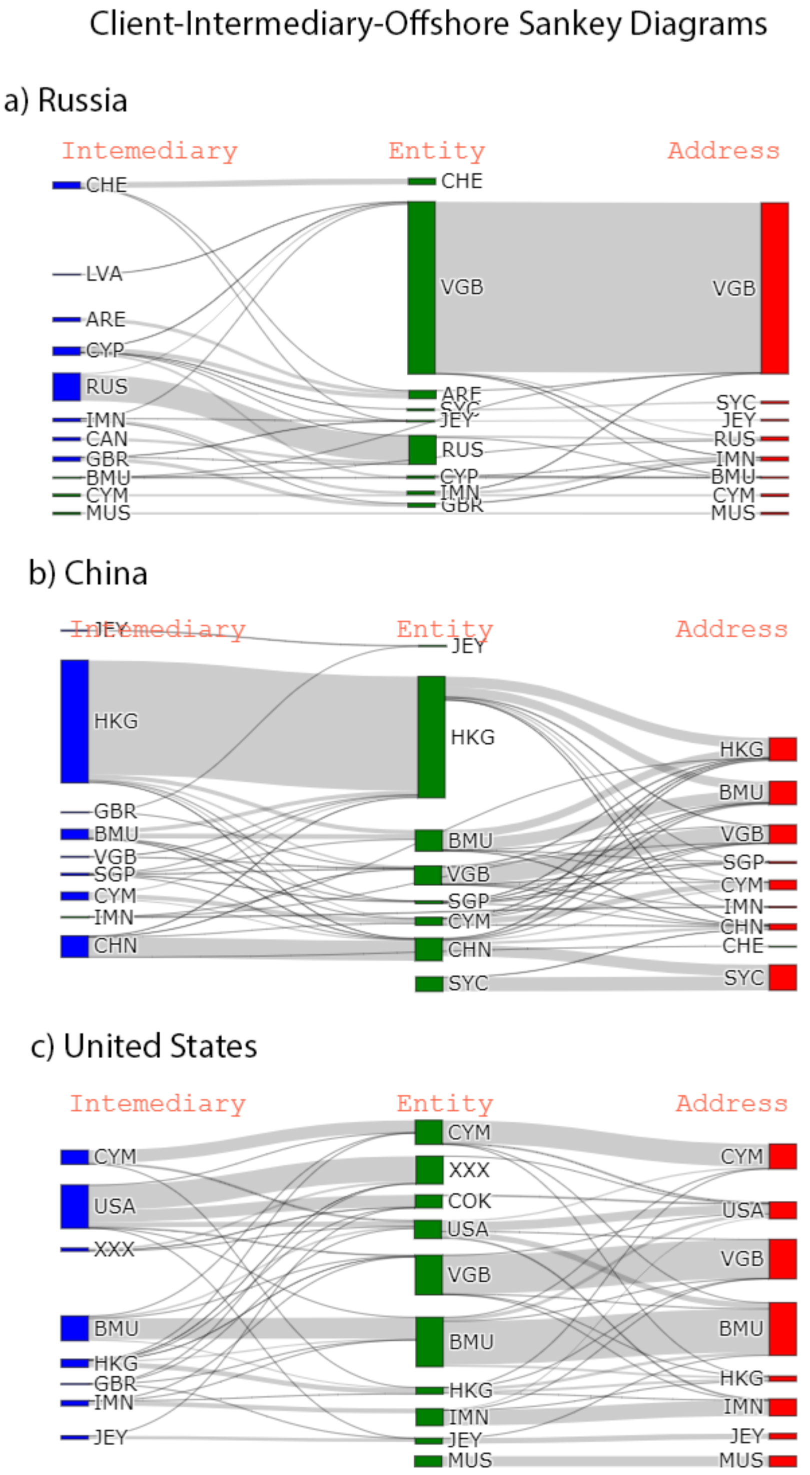

Fig. S1. Sankey Diagrams by clients from each country, specifying their choice of intermediary, entity location, and offshore location.

3. Powerlaw extends to generate bipartite graph

We validate the power-law by including all nodes in the intermediary-client bipartite graph.

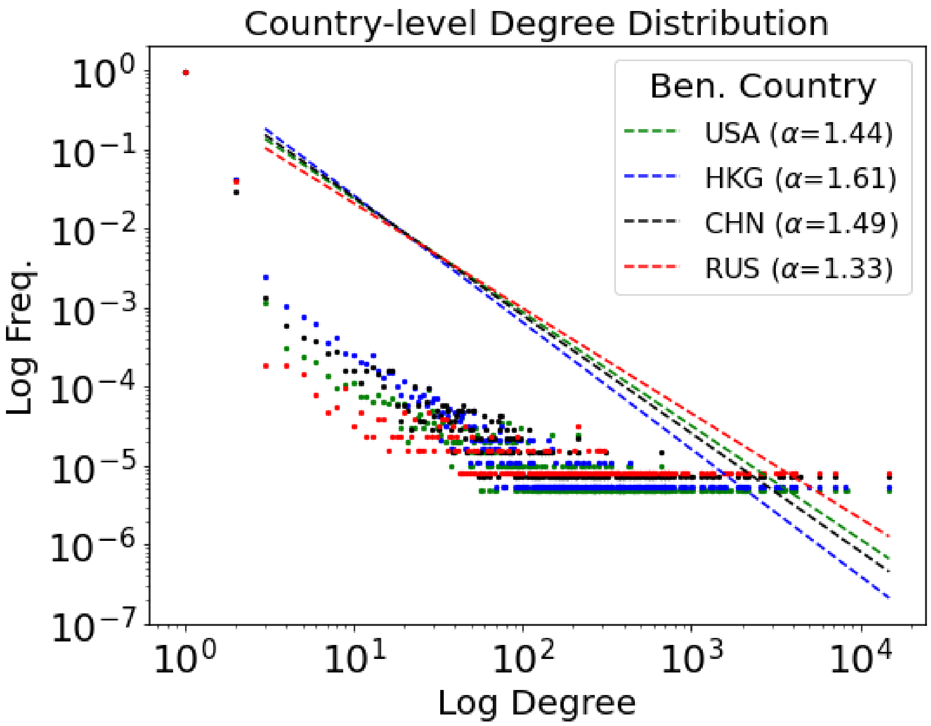

Fig. S2. Degree distribution of full bipartite graph (both intermediaries and clients), by country.

4. Knock-out top intermediaries

Country	Intermediary 1	Intermediary 2	Intermediary 3
RUS	LEGAL CONSULTING SERVICES LIMITED	Unitrust Corporate Services Ltd.	CONSULCO INTERNATIONAL LIMITED
USA	Appleby Services (Bermuda) Ltd.	Appleby Trust (Cayman) Ltd.	KHD HUMBOLDT WEDAG INTERNATIONAL LTD.
CHN	ORION HOUSE SERVICES (HK) LIMITED	OFFSHORE BUSINESS CONSULTANT (INT'L) LIMITED	Offshore Business Consultant (HK) Ltd.
HKG	Company Kit Limited	MUFG Fund Services (Bermuda) Limited	Ready-Made Registrations Limited

Table S2. Top intermediaries that were knocked out in the simulated experiment.

5. Power-law Fits

Log-likelihood comparisons across different possible distributions. One note is while lognormal distributions seem to fit better for China and Russia, this is in part due to their heavier-tail and also due to the distribution being discrete with a lower-bound. It was shown early on that lognormal distributions with a lower-bound converge to a power-law (1, 2).

Distrib 1	Distrib 2	Log Likelihood	P-value
power-law	expontential	1134.0718	9.70E-17
power-law	expontential	1359.832	3.40E-11
power-law	expontential	322.56539	3.67E-06
power-law	expontential	218.01097	2.34E-05
power-law	lognormal	265.09519	2.58E-06
power-law	lognormal	7.1313864	8.17E-2
power-law	lognormal	-55.32297	1.57E-15
power-law	lognormal	-30.03486	9.19E-13

Table S3. Log-likelihood evaluations across different long-tail fits.

References

1. DG Champernowne, A model of income distribution. *The Econ. J.* **63**, 318–351 (1953).
2. WJ Reed, The pareto, zipf and other power laws. *Econ. letters* **74**, 15–19 (2001).
